# Systematic review and meta‑analysis of factors predicting postoperative lung function after lung cancer resection

**DOI:** 10.20452/wiitm.2024.17892

**Published:** 2024-07-31

**Authors:** Hongling Wang, Lihong He, Xiaoyun Hu, Gongxue Xian

**Affiliations:** Department of Interventional Surgery, Yantaishan Hospital, Yantai City, China; Outpatient Department, The General Hospital of Western Theater Command of Chinese People’s Liberation Army, Chengdu, Sichuan, China; Operating room, Hai’an Traditional Chinese Medicine Hospital, Hai’an, Jiangsu, China

**Keywords:** computed tomography perfusion, computed tomography volume and density, lobectomy, lung cancer, postoperative pulmonary function

## Abstract

**INTRODUCTION:**

Lung resection continues to be the most effective treatment for early‑stage lung cancer. Prediction of postoperative lung function is particularly important when evaluating patient eligibility for surgery, as it helps assess the likelihood of experiencing difficulty breathing after the operation.

**AIM:**

We aimed to identify the most common methods used to predict postoperative lung function in clinical practice and to compare their accuracy.

**MATERIALS AND METHODS:**

A systematic review and meta‑analysis were performed to synthesize research focused on the prediction of postoperative lung function. A total of 10 studies were included in the analysis. The Cochrane risk of bias tool was utilized to evaluate the risk of bias in the studies. Additionally, a meta‑analysis of the mean difference between the predicted and measured values of forced expiratory volume in 1 second (FEV_1_) was conducted. The I^2 ^value was computed as a metric of coherence among studies, while funnel plots and the Begg test were used to evaluate the likelihood of publication bias.

**RESULTS:**

The analyzed studies had a low risk of bias. The meta‑analysis showed that computed tomography (CT) volume and density measurement had the highest level of accuracy for predicting postoperative FEV_1 _, with a mean difference between the predicted and actual value of 83 ml (95% CI, 41–116).

**CONCLUSIONS:**

The results indicate that using CT volume and density is the optimal method for predicting postoperative FEV_1 _. Additional research is necessary to establish the connection between the type of surgical procedure, adopted thresholds, and outcomes reported by patients.

## INTRODUCTION 

Non–small cell lung cancer (NSCLC) is a collective term that refers to several types of lung malignancies, such as squamous cell carcinoma and adenocarcinoma, which share comparable characteristics and behaviors.^1^^; ^^2^ The symptoms encompass chronic cough, respiratory distress, involuntary weight loss, and hemoptysis.^3^ Primary therapeutic interventions include surgery, chemotherapy, and radiation treatment.^4^ Surgical excision with curative intent is the recommended therapy for stage I and II NSCLC.^5^ The 5‑year survival rate for regional NSCLC that has spread to nearby tissues or lymph nodes is 35%. In contrast, the 5‑year survival rate for metastatic NSCLC is 7%.^6^ Patients referred for invasive treatment require a comprehensive preoperative assessment. It involves evaluation of the transfer factor for carbon monoxide (TL_CO_), also known as diffusing capacity, which is a pulmonary function test that measures the capacity of the lung to absorb oxygen from breathed air,^7^ and forced expiratory volume in 1 second (FEV_1_), which refers to the greatest amount of air that an individual may forcibly exhale during the first second after taking a deep breath.^8^ The predicted postoperative values of FEV_1_ and TL_CO_ are also used to estimate the risk of mortality and impaired lung function after surgery, with a percentage of predicted value in the range of 30% to 60% indicating that a patient requires further evaluation to confirm their eligibility for surgery.^9^^; ^^10^

To date, the accuracy and precision of the many methods that are purported to predict postoperative lung function have not been systematically assessed. Prediction plays a crucial role in guiding important therapeutic decisions, such as whether to proceed with surgical resection or opt for other treatment methods (eg, chemotherapy or radiation). When curative medical therapies are insufficient, surgical resection remains the primary method of treatment for cancer patients. Its aim is to achieve a complete resection with secure margins by removing the affected tissue, a portion, or the entirety of an organ. Lung resection is a surgical procedure used to treat pulmonary infections, emphysema, bronchiectasis, or cancer by removing the entire lung or its portion. It can be performed using minimally invasive techniques or via open surgery. In contrast, chemotherapy employs cytotoxic pharmaceuticals administered intravenously to selectively eliminate cancer cells from various anatomical sites, whereas radiation therapy utilizes high‑energy beams (eg, X‑rays or proton therapy) to specifically target and eradicate cancer cells at the site of the tumor.^11^^; ^^12^ Patient counseling entails making predictions regarding the operative risk, and a comprehensive analysis has shown that a significant proportion of patients with resectable malignancies do not view resection as their preferred treatment option.^13^^; ^^14^ Moreover, predictions of a high operative risk might potentially lead to postponement of therapy in order to accommodate further investigations, such as evaluation of cardiopulmonary endurance.^15^^; ^^16^ Therefore, it is necessary to explicitly compare the methods used for prediction.

## AIM 

The purpose of this study was to identify the optimal strategy for the prediction of postoperative lung function following lung cancer resection by a systematic review and meta‑analysis of relevant observational studies ^17^^; ^^18^^; ^^19^^; ^^20^^; ^^21^^; ^^22^^; ^^23^^; ^^24^^; ^^25^^; ^^26^ chosen based on predefined inclusion exclusion criteria.

## MATERIALS AND METHODS 

Following the guidelines defined in the Preferred Reporting Items for Systematic Reviews and Meta‑Analyses (PRISMA) statement,^27^ this study encompassed a systematic review and meta‑analysis of factors employed to predict postoperative lung function in patients undergoing lung cancer resection.

All procedures performed in the study were in accordance with the institutional and / or national research committee standards and with the 1964 Helsinki Declaration and its later amendments, or comparable ethical standards.

### Search strategy and study selection 

The present meta‑analysis was conducted following a comprehensive search of major databases, including Embase, PubMed, Scopus, and the Cochrane Library. The search approach incorporated key words related to lung surgery, lung function measurement, postoperative period, and prediction or correlation. The following search terms were used: *thoracic surgery, lung cancer, lung function, postoperative lung function, lung cancer resection, computed tomography or CT, CT volume and density, forced expiratory volume in 1 s or FEV_1_, lobectomy, pneumonectomy, perfusion scintigraphy, CT perfusion, single photon emission computed tomography or SPECT, segment counting, mean difference, ventilation scintigraphy, meta‑analysis, and systematic review and meta‑analysis*. Following the Population, Intervention, Comparison, Outcomes and Study (PICOS) criteria, we identified and assessed the key words for their agreement in both the Embase and Medline databases (TABLE 1). The specified key words were entered into the title / abstract / keyword field during the Scopus search. In the Cochrane database, we searched for papers related to postoperative lung function, prediction techniques, and lung cancer resection. 

Using predetermined search criteria, we aimed to identify studies that evaluated the accuracy of various predictive factors in assessing postoperative pulmonary function in patients diagnosed with lung cancer, as measured by the standard mean difference between the predicted and measured FEV_1 _values. The criteria for study selection were based on the PICO framework^28^ (Population referred to individuals diagnosed with lung cancer; Intervention referred to prediction methods; Comparison represented the control, and Outcomes referred to standard mean difference between the measured and projected FEV_1 _value. Only observational studies were considered for analysis. There were no restrictions or limitations with respect to the language, date of publication, or any other study feature. To identify the pertinent publications, 2 investigators (LH and QZ) conducted a comprehensive individual review of the whole corpus of relevant scientific literature. The same 2 investigators conducted a separate analysis of titles and abstracts of the identified publications to find all that potentially met the criteria for inclusion, and independently assessed their whole content to ascertain suitability. Disputes over eligibility were resolved by consensus, with the inclusion of a third reviewer, if necessary. Studies that were assessed to have a minimal risk of bias were included in the meta‑analysis.

### Inclusion and exclusion criteria 

The inclusion criteria were as follows: 1) study population comprising adults with a suspected or confirmed diagnosis of primary lung cancer; 2) the procedure performed was lung resection with curative intent (pneumonectomy, lobectomy, segmentectomy, or wedge resection); and 3) lung function assessment was performed before and after the operation using at least 2 methods to predict post-operative lung function and evaluate the accuracy of the predictions. Studies that included both patients with benign and malignant tumors were considered suitable if a majority of the participants had primary lung cancer. Studies involving surgical procedures related to benign cancer, palliative care, diagnostic purposes, emergencies, and bronchoscopy alone were excluded.

**TABLE 1:** Database search strategy

Database	Search strategy
Scopus	1. *Thoracic surgery* OR l*ung cancer *OR l*ung function *OR *postoperative lung function *OR* lung cancer resection* OR *computed tomography* OR *CT* OR *CT* *volume and density *OR *forced expiratory volume in 1 s *OR *FEV_1_* OR *lobectomy *OR* pneumonectomy *OR *perfusion scintigraphy* OR *CT perfusion*
	2. *Single photon emission computed tomography* OR* SPECT OR segment counting* OR *mean difference *OR *ventilation scintigraphy *OR* meta­‐analysis *OR *systematic review and meta­‐analysis*
	3. #1 AND #2
PubMed	1. *Thoracic surgery *OR *lung cancer* OR *postoperative lung function *(MeSH terms) OR* lung function* OR *lung cancer resection *OR *computed tomography *OR *CT* (all fields) OR *CT volume and density* OR *forced expiratory volume in 1 s *(all fields) OR *FEV_1_* OR *lobectomy* (all fields) OR *pneumonectomy* (all fields) OR *perfusion scintigraphy* (all fields) OR *CT perfusion* (all fields)
	2. *Single photon emission computed tomography *OR *SPECT *OR *segment counting* (MeSH terms) OR *mean difference *OR *systematic review and meta­‐analysis* OR *meta­‐analysis* (all fields)
	3. #1 AND #2
Embase	1.* Thoracic surgery* / exp OR *lung* *cancer* / exp OR *postoperative lung function* / exp OR *lung function* / exp OR* lung cancer resection* / exp OR *computed tomography* / exp OR *CT* / exp OR *CT* *volume and density* / exp OR *forced expiratory volume in 1 s* / exp OR *FEV_1_* / exp OR *lobectomy* / exp OR *pneumonectomy* / exp OR* Perfusion scintigraphy */ exp OR *CT perfusion */ exp
	2. *Single photon emission computed tomography* / exp OR *SPECT* / exp OR* segment counting* / exp OR *mean difference* / exp OR *meta­‐analysis* / exp OR *systematic review and meta­‐analysis*
	3. #1 AND #2
Cochrane library	1. *Thoracic surgery*: ti, ab, kw OR *lung cancer*: ti, ab, kw OR *postoperative lung* *function*: ti, ab, kw OR *lung* *function*: ti, ab, kw OR *lung cancer resection*: ti, ab, kw OR *computed tomography *OR *CT volume and density*: ti, ab, kw OR *forced expiratory volume in 1 s*: OR FEV_1_: ti, ab, kw OR *lobectomy*: ti, ab, kw OR *pneumonectomy*: ti, ab, kw OR *perfusion scintigraphy*: ti, ab, kw OR *CT perfusion (word variations have been searched)*
	2. *Single photon emission computed tomography*: ti, ab, kw OR *SPECT*: ti, ab, kw OR *segment* *counting*: ti, ab, kw *or mean* *difference*: ti, ab, kw OR *meta­*‐*analysis*: ti, ab, kw OR* systematic review* *and meta­‐analysis (word variations have been searched)*
	3. #1 AND #2

Abbreviations: exp, explosion in Emtree—searching of selected subject terms and related subjects; MeSH, Medical Subject Headings; ti, ab, kw, title,

abstract, or key word field

### Risk of bias evaluation 

The assessment of bias was conducted using a Cochrane Collaboration approach outlined in the Cochrane Handbook (version 5.3).^29^ The instrument has 7 components, including one that evaluates bias resulting from confounding. This study analyzed the following types of bias: measurement bias in assessing exposure, bias in selecting participants for the study, bias resulting from treatments after exposure, bias due to missing data, measurement bias in assessing the outcome, and bias in selecting the reported results. Two reviewers (LH and QZ) undertook an impartial evaluation to detect any potential bias. WL, who served as an arbitrator, was responsible for resolving any outstanding issues. Ultimately, the possible bias was assessed and categorized as either “uncertain risk,” “high risk,” or “low risk.”

### Data management and evidence synthesis 

For the purpose of documenting extracted data from eligible studies and risk of bias assessment, a pre‑designed electronic form was utilized. The extracted information comprised the following: study authors and year of publication, publishing journal, country where the study was conducted, total participant count, sample size, participant age and sex, procedure type, predictive factors, time to postoperative lung function assessment, primary outcomes, and statistical analysis. One investigator performed independent data extraction, while a second one verified the data. Any inconsistencies were handled by consensus with a third investigator, if needed. The primary summary measurements included the mean difference between measured and predicted postoperative lung function and the standard deviation of the mean difference. Meta‑analysis of standard mean difference was performed using the generic inverse variance method in RevMan software, version 5.4,^30^ and forest plots ^31^ were created to evaluate the influence of outcome drivers. The I^2^ value^32^ was calculated as a measure of consistency across studies, and funnel plots^33^ and the Begg test ^34^ were used to assess the risk of publication bias.

## RESULTS

### Included studies

**FIGURE 1:**
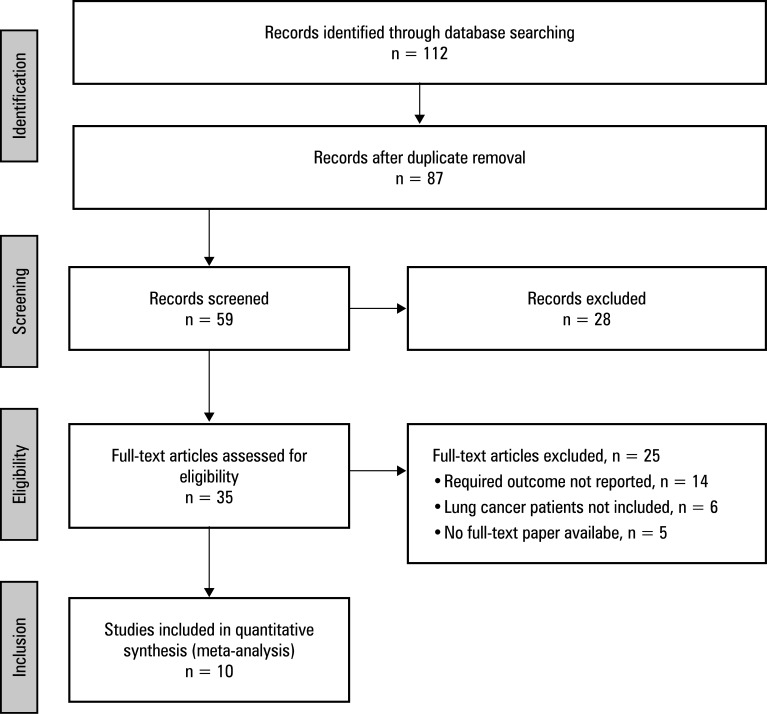
Preferred Reporting Items for Systematic Reviews and Meta-Analyses (PRISMA) study flow diagram

The search was conducted via inclusive computeraided scanning of numerous databases, yielding 112 publications that met the PICOS inclusion‑exclusion criteria. A total of 25 items were excluded due to duplication, leaving 87 articles. Subsequently, 28 items were removed owing to irrelevant titles and abstracts, whereas 59 articles were further examined. Following screening, 35 records were assessed for eligibility. Of them, 24 were removed because they did not meet the inclusion criteria, had insufficient data to enable generation of 2 × 2 tables, or lacked important outcome measures. Finally, 10 observational studies published between 2000 and 2023 were included in the meta‑analysis (FIGURE 1). All of them were performed on lung cancer patients of various ages. Main characteristics of the included studies are shown in TABLE 2 .

### Risk of bias assessment 

**FIGURE 2:**
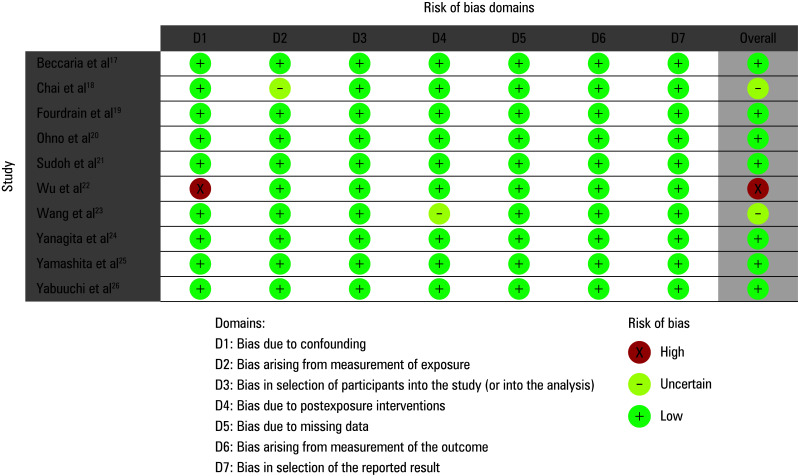
Traffic light plot for risk of bias assessment

**FIGURE 3:**
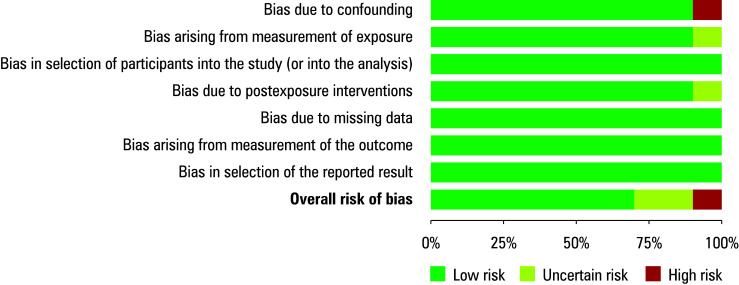
Risk of bias summary plot

**FIGURE 4:**
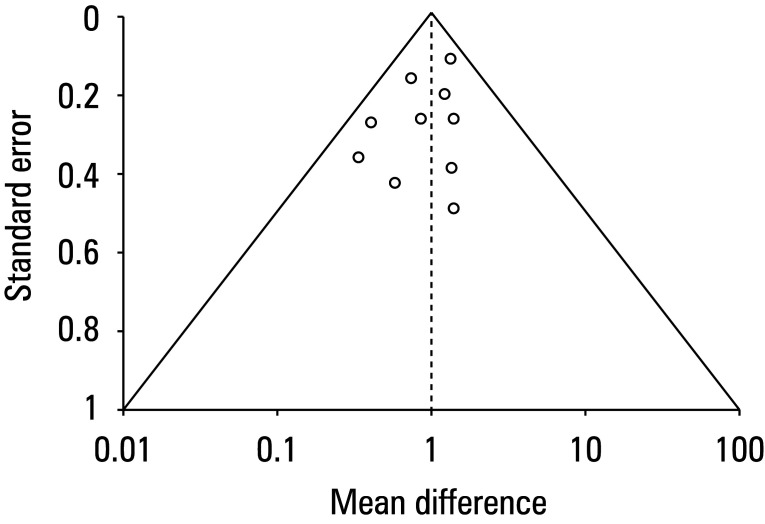
Funnel plot for publication bias

The studies included were assessed for potential risk of bias using a predesigned questionnaire. The overall risk of bias was evaluated as low (FIGURE 2 and FIGURE 3). Among the 10 observational studies analyzed, 7 were found to have a low risk of bias, while 2 were identified as having a moderate risk of bias related to the measurement of exposure^18^ or postexposure interventions.^23^ Only 1 study^22^ was found to have a high risk of bias due to confounding factors. Minimal risk of publication bias was further confirmed by the symmetrical funnel plot shown in FIGURE 4 and the *P value *of the Begg test indicating a lack of significance (*P* = 0.2).

### Quantitative synthesis: meta-analysis of mean difference between predicted and observed postoperative lung function 

**FIGURE 5:**
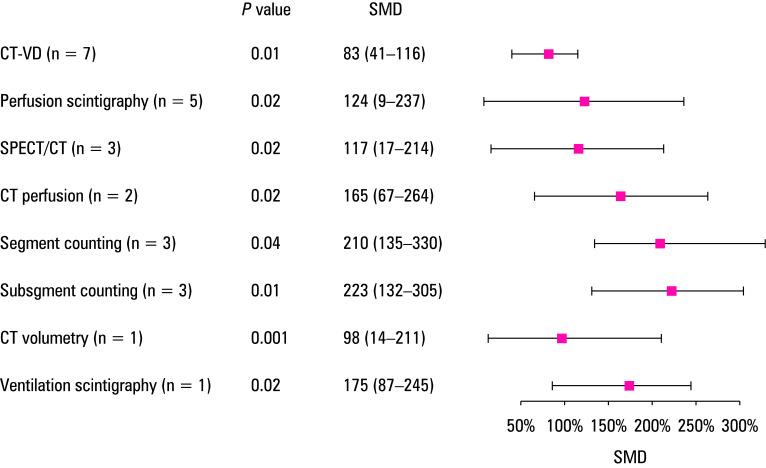
Forest plot for standard mean difference (SMD) between predicted and measured values of forced expiratory volume in 1 second assessed using different prediction methods Abbreviations: see TABLE 2 and TABLE 3

The meta‑analysis presents the findings of studies in which postoperative FEV_1 _value was predicted using various methods. The results are shown in TABLE 3 and the related forest plots are displayed in FIGURE 5 . The approach involving measurement of computed tomography volume and density (CT‑VD) demonstrated the highest accuracy, with a mean FEV_1 _difference of 83 ml (95% CI, 41–116). The precise threshold for the minimum clinically significant change in FEV_1 _following surgery is not exactly known; however, it has been determined to be 100 ml in the context of chronic obstructive pulmonary disease.^35^^; ^^36^ Therefore, any difference in FEV_1 _between the predicted actual values that is smaller than 100 ml should not be perceptible from a clinical standpoint.^37^^; ^^38^ Hence, the data indicate that CT‑VD is the preferred method for predicting postoperative FEV_1 _. The level of heterogeneity was low for CT‑VD, moderate for SPECT‑CT and CT perfusion imaging, and high for segment counting, subsegment counting, and perfusion scintigraphy (TABLE 3).

## DISCUSSION 

Maintaining optimal lung function postsurgery is crucial, and fortifying the respiratory muscles both before and after the procedure can effectively lower the likelihood of contracting a pulmonary infection by 50%, as well as mitigate the danger of other complications, such as lung collapse, diminished lung capacity, and impaired mucus clearance from the respiratory system.^39^^; ^^40^ Multiple variables influence the restoration of pulmonary function following lung cancer surgery.^41^^; ^^42^ The selected surgical technique and the extent of lung tissue removal are significant determinants of pulmonary function loss. Due to the requirement for larger incisions and extended hospital stay, open surgery is commonly considered less favorable than a minimally invasive approach in certain surgical fields, such as colon and pulmonary surgery.^43^^; ^^44^^; ^^45^ The benefits of minimally invasive surgical techniques include less discomfort, accelerated recovery, and faster resumption of work. Minimally invasive techniques eliminate the need for extensive tissue removal by utilizing tiny devices and cameras, and result in reduced discomfort and faster healing, with reduced duration of postsurgery suffering and pain. ^46^

**TABLE 2:** Brief summary of the included studies

Study	Journal of publication	Country of study	Total number of participants	Sample size, n	Age of participants, y	Sex (M/F)	Procedure	Prediction technique	Time to postoperative lung function assessment, mo	Primary outcomes	Statistical analysis
Beccaria et al^17^	Chest	Italy	93	62	62	77/16	Lobectomy & pneumonectomy	Segment counting, CT‑VD	6	FEV_1_	MD, correlation
Chae et al^18^	Investigative Radiology	Korea	67	51	64	48/16	Lobectomy & pneumonectomy	Perfusion scintigraphy, CT perfusion, CT­‐VD	6	FEV_1_	MD, correlation
Fourdrain et al^19^	Journal of Thoracic Disease	France	37	23	61	32/5	Lobectomy & pneumonectomy	Segment counting, subsegment counting. perfusion scintigraphy, ventilation scintigraphy, CT­‐VD	3	FEV_1_	MD, correlation
Ohno et al^20^	Journal of Magnetic Resonance Imaging	Japan	60	70	71	30/30	Lobectomy & pneumonectomy	Perfusion scintigraphy, ventilation scintigraphy, PECT, SPECT/CT	6	FEV_1_	MD, correlation
Sudoh et al^21^	Journal of Thoracic and Cardiovascular Surgery	Japan	22	22	71	19/3	Lobectomy & segmentectomy	Subsegment counting, SPECT/CT	4	FEV_1_	MD, correlation
Wu et al^22^	American Journal of Roentgenoloy	Taiwan	52	34	69	42/10	Lobectomy & segmentectomy	Perfusion scintigraphy, CT­‐VD	3	FEV_1_	MD, correlation
Wang et al^23^	Chest	Canada	57	28	65	35/22	Segmentectomy, lobectomy & pneumonectomy	Segment counting, CT­‐VD	12	FEV_1_	MD, correlation
Yanagita et al^24^	Japanese Journal of Radiology	Japan	34	30	70	20/10	Lobectomy & pneumonectomy	SPECT, CT	6	FEV_1_	MD, correlation
Yamashita et al^25^	Academic Radiology	Canada	25	14	65	37/28	Lobectomy & pneumonectomy	Perfusion scintigraphy, CT perfusion, CT­‐VD	3	FEV_1_	MD, correlation
Yabuuchi et al^26^	European Journal of Radiology	Japan	49	49	67	26/23	Lobectomy	Subsegment counting, CT volumetry, CT­‐VD	6	FEV_1_	MD, correlation

Abbreviations: CT, computed tomography; CT­‐VD, computed tomography volume and density; F, female; FEV_1_, forced expiratory volume in 1 second; M, male; MD, mean difference; SPECT, single photon emission computed tomography

**TABLE 3:** Meta‑analysis of factors predicting postoperative lung function

Prediction technique	Number of studies	SMD^a^, ml	95% CI	*P *value	Heterogeneity (I^2^)
CT‑VD	7	83	41–116	0.01	21
Perfusion scintigraphy	5	124	9–237	0.02	78
SPECT/CT	3	117	17–214	0.015	41
CT perfusion	2	165	67–264	0.024	34
Segment counting	3	210	135–330	0.035	71
Subsegment counting	3	223	132–305	0.014	67
CT volumetry	1	98	14–211	0.001	–
Ventilation scintigraphy	1	175	87–245	0.021	–

a Difference between predicted and measured value of forced expiratory volume in 1 second; Abbreviations: SMD, standard mean difference; others, see TABLE 2

Predicting postoperative lung function is crucial during preoperative assessment of patients diagnosed with lung cancer. In order to thoroughly evaluate the risk of mortality and breathing difficulties following surgery, a full preoperative examination is conducted, which includes an evaluation of the predicted postoperative FEV_1_.^47^^; ^^48^ This parameter reflects the maximum quantity of air that a person can forcefully exhale within the first second after taking a deep breath.^49^ If the predicted value falls between 30% and 60%, it indicates the need for additional patient evaluation.^50^

Various methods can be used to assess the average difference in FEV_1 _for predicting postoperative lung function, including CT techniques such as volume and density analysis, perfusion scintigraphy, SPECT‑CT, CT perfusion, segment and subsegment counting, CT volumetry, and ventilation scintigraphy.^51^^; ^^52^^; ^^53^^; ^^54^ The observational studies included in this analysis compared various prediction methods and presented their respective findings. For instance, Beccaria et al^17^ found that a predicted postoperative FEV_1 _value of 40% or more may effectively identify individuals that do not need additional testing and are not at a risk of long‑term respiratory dysfunction. 

Chae et al^18^ used the Bland–Altman plots to determine the limits of agreement between the measured the projected postoperative FEV_1 _values. They showed that for scintigraphy, the limits of agreement ranged from –29.3% to 26.9%, while for CT, the range was from –28.9% to 17.3%. The error rate of CT was similar to that of scintigraphy (15.4% vs 17.8%). Dual‑energy perfusion CT demonstrated superior accuracy, as compared with perfusion scintigraphy, in predicting postoperative lung function. According to Fourdrain et al,^19^ quantitative CT imaging seems to be an acceptable technique for evaluating the predicted postoperative FEV_1 _. It also appears to be more dependable than other methods. The estimation of predicted postoperative FEV_1 _value, as a component of the preoperative evaluation, does not require any further morphologic examinations, namely scintigraphy. This approach has the potential to become the standard way for evaluating predicted postoperative FEV_1 _. 

Ohno et al^20^ concluded that SPECT‑CT using ^81m^krypton and ^99m^technetium‑labeled macroaggregated albumin demonstrated superior reproducibility and accuracy in predicting postoperative lung function, as compared with SPECT and planar imaging. Sudoh et al^21^ reported that combining SPECT‑CT images acquired during breath‑holding enables precise estimation of postoperative pulmonary function. However, this approach did not demonstrate statistical advantage over the simpler segment‑counting method. According to Wu et al,^22^ both quantitative CT and perfusion scintigraphy accurately predicted postoperative FEV_1 _value in patients undergoing pneumonectomy (n = 28; r = 0.88 vs r = 0.86, respectively;* P* <0.001) and lobectomy (n = 16; r = 0.9 vs r = 0.8, respectively;* P* <0.001). The Bland–Altman plot indicated a high level of concordance between the 2 methods. Quantitative CT is commonly utilized for predicting postoperative FEV_1 _evaluation due to its simplicity. In a study by Wang et al,^23^ a notable decrease in FEV_1 _values evaluated with this method was found after lung resection.

Yanagita et al^24^ noted a substantial difference between the predicted and actual values of vital capacity, forced vital capacity, and FEV_1 _(R^2 ^= 0.56–0.77; *P* <0.001), as determined using CT volumetry imaging. In turn, Yamashita et al^25^ showed that CT subtraction imaging is as precise as radioisotope perfusion scintigraphy in predicting postoperative lung function. This finding is particularly relevant for the preoperative evaluation of resectable lung cancer in high‑risk patients. In their study, Yabuuchi et al^26^ discovered that analyzing the volume of the lungs using inspiratory / expiratory CT data can be a valuable method for predicting postoperative pulmonary function following lobectomy for primary lung cancer.

The latest British recommendations published in 2017^55^ indicate that patients should have a CT scan performed at least twice before resection. This includes an initial diagnostic scan and a second one performed for staging. Positron emission tomography / CT is an attractive approach toward predicting postoperative lung function since it uses real‑time imaging without the need for additional sessions and delays.^56^ Moreover, CT densitometry has demonstrated superiority over spirometry in the prediction of pulmonary problems, such as extended air leak following lung resection, as well as in identification of individuals who are at a higher risk of experiencing these difficulties.^57^^; ^^58^^; ^^59^ Patients are eligible for resection without the need for additional evaluation if their FEV_1_ and TL_CO_ values are greater than or equal to 60% of the predicted values, as per the guidelines established by the American College of Chest Physicians.^5^ Furthermore, quantitative measures of lung volume and density acquired from preoperative CT scans correlate with pulmonary function test results, enabling the prediction of pulmonary function in patients diagnosed with lung cancer and identification of individuals who are amenable to surgical resection.^60^ In a similar vein, our meta‑analysis showed that prediction of FEV_1 _after lung resection is the most accurate and precise when performed based on combined CT‑VD, and the accuracy of other evaluated methods for predicting postoperative FEV_1 _is low. These findings are especially pertinent to future guidelines for the evaluation of eligibility for lung cancer surgery. A consolidated CT‑based risk assessment that predicts both postoperative pulmonary function and the likelihood of post-operative pulmonary problems would be a useful tool for clinicians.

### Strengths and limitations 

This systematic review and meta‑analysis aimed to identify the most effective method for predicting postoperative lung function following lung cancer resection. Precise search criteria were adopted to allow for identification of studies investigating postoperative lung function, prediction techniques, and lung cancer resection across several databases. A notable advantage is that our findings have worldwide relevance, as the included studies were conducted in different countries and covered a wide variety of clinical settings. They were also heterogenous with respect to the surgical procedures, time of follow‑up lung function evaluation, and specific features of the prediction method. Nevertheless, it is crucial to acknowledge certain limitations. Firstly, only a small proportion of the identified studies were included in the final analysis, which may have led to biased results. Moreover, due to a limited number of patients in each of the included studies, further investigation is necessary to establish a precise correlation between the type of surgical procedure, FEV_1 _thresholds used, and patient outcomes.

## CONCLUSIONS 

In conclusion, CT‑VD is the most precise and accurate method for predicting post‑ operative FEV_1 _. Nevertheless, further investigations involving a greater number of studies are required to substantiate these findings, establish a correlation between the surgical methods, adopted thresholds, and patient‑reported outcomes, and strengthen the existing body of evidence.

## References

[BIBR-1] Gridelli C., Rossi A., Carbone D.P. (2015). Non‐small‐cell lung cancer. Nat Rev Dis Primers.

[BIBR-2] Alduais Y., Zhang H., Fan F. (2023). Non‐small cell lung cancer (NSCLC): a review of risk factors, diagnosis, and treatment. Medicine (Baltimore.

[BIBR-3] Xing P.Y., Zhu Y.X., Wang L. (2019). What are the clinical symptoms and physical signs for non‐small cell lung cancer before diagnosis is made? A nation‐wide multicenter 10‐year retrospective study in China. Cancer Med.

[BIBR-4] Muers M.F., Round C.E. (1993). Palliation of symptoms in non‐small cell lung cancer: a study by the Yorkshire Regional Cancer Organisation Thoracic Group. Thorax.

[BIBR-5] Howington J.A., Blum M.G., Chang A.C. (2013). Treatment of stage I and II non‐small cell lung cancer: diagnosis and management of lung cancer. Chest.

[BIBR-6] Lemjabbar‐Alaoui H., Hassan O.U., Yang Y.W., Buchanan P. (2015). Lung cancer: biology and treatment options. Biochim Biophys Acta.

[BIBR-7] Hegewald M.J. (2009). Diffusing capacity. Clin Rev Allergy Immunol.

[BIBR-8] Menezes A.M., Pérez‐Padilla R., Wehrmeister F.C. (2014). FEV1 is a better predictor of mortality than FVC: the PLATINO cohort study. PLoS One.

[BIBR-9] Roy P.M. (2018). Preoperative pulmonary evaluation for lung resection. J Anaesthesiol Clin Pharmacol.

[BIBR-10] Kearney D.J., Lee T.H., Reilly J.J. (1994). Assessment of operative risk in patients undergoing lung resection. Importance of predicted pulmonary function. Chest.

[BIBR-11] Debela D.T., Muzazu S.G., Heraro K.D. (2021). New approaches and procedures for cancer treatment: current perspectives. SAGE Open Med.

[BIBR-12] Anand U., Dey A., Chandel A.K.S. (2022). Cancer chemotherapy and beyond: current status, drug candidates, associated risks and progress in targeted therapeutics. Genes Dis.

[BIBR-13] Kwaśniewska D., Fudalej M., Nurzyński P. (2023). How a patient with resectable or borderline resectable pancreatic cancer should be treated – a comprehensive review. Cancers (Basel.

[BIBR-14] Hewitt D.B., Brown Z.J., Pawlik T.M. (2022). Current perspectives on the surgical management of perihilar cholangiocarcinoma. Cancers (Basel.

[BIBR-15] Upadhyay R.K. (2015). Emerging risk biomarkers in cardiovascular diseases and disorders. J Lipids.

[BIBR-16] Bosscher R, C Dausin, P Claus (2021). Endurance exercise and the risk of cardiovascular pathology in men: a comparison between lifelong and late‐onset endurance training and a non‐athletic lifestyle – rationale and design of the Master@Heart study, a prospective cohort trial. BMJ Open Sport Exerc Med.

[BIBR-17] Beccaria M., Corsico A., Fulgoni P. Lung cancer resection: the prediction of postsurgical outcomes should include long‐term functional results. Chest.

[BIBR-18] Chae E.J., Kim N., Seo J.B. (2013). Prediction of postoperative lung function in patients undergoing lung resection: dual‐energy perfusion computed tomography versus perfusion scintigraphy. Invest Radiol.

[BIBR-19] Fourdrain A., Dominicis F., Lafitte S. (2017). Quantitative computed tomography to predict postoperative FEV1 after lung cancer surgery. J Thorac Dis.

[BIBR-20] Ohno Y., Koyama H., Takenaka D. (2007). Coregistered ventilation and perfusion SPECT using krypton‐81m and Tc‐99m‐labeled macroaggregated albumin with multislice CT utility for prediction of postoperative lung function in non‐small cell lung cancer patients. Acad Radiol.

[BIBR-21] Sudoh M., Ueda K., Kaneda Y. (2006). Breath‐hold single‐photon emission tomography and computed tomography for predicting residual pulmonary function in patients with lung cancer. J Thorac Cardiovasc Surg.

[BIBR-22] Wu M.T., Pan H.B., Chiang A.A. (2002). Prediction of postoperative lung function in patients with lung cancer: comparison of quantitative CT with perfusion scintigraphy. AJR Am J Roentgenol.

[BIBR-23] Wang J.S., Abboud R.T., Wang L.M. (2006). Effect of lung resection on exercise capacity and on carbon monoxide diffusing capacity during exercise. Chest.

[BIBR-24] Yanagita H., Honda N., Nakayama M. (2013). Prediction of postoperative pulmonary function: preliminary comparison of single‐breath dual‐energy xenon CT with three conventional methods. Jpn J Radiol.

[BIBR-25] Yamashita C.M., Langridge J., Hergott C.A. (2010). Predicting postoperative FEV1 using spiral computed tomography. Acad Radiol.

[BIBR-26] Yabuuchi H., Kawanami S., Kamitani T. (2016). Prediction of post‐operative pulmonary function after lobectomy for primary lung cancer: a comparison among counting method, effective lobar volume, and lobar collapsibility using inspiratory / expiratory CT. Eur J Radiol.

[BIBR-27] Liberati A., Altman D.G., Tetzlaff J. (2009). The PRISMA statement for reporting systematic reviews and meta‐analyses of studies that evaluate healthcare interventions: explanation and elaboration. BMJ.

[BIBR-28] Cumpston M.S., McKenzie J.E., Thomas J., Brennan S.E. (2020). The use of ‘PICO for synthesis’ and methods for synthesis without meta‐analysis: protocol for a survey of current practice in systematic reviews of health interventions. F1000Res.

[BIBR-29] Higgins J.P., Altman D.G., Gøtzsche P.C. (2011). Cochrane Bias Methods Group; Cochrane Statistical Methods Group. The Cochrane Collaboration’s tool for assessing risk of bias in randomised trials. BMJ.

[BIBR-30] Schmidt L., Shokraneh F., Steinhausen K., Adams C.E. (2019). Introducing RAPTOR. RevMan Parsing Tool for Reviewers. Syst Rev.

[BIBR-31] Dettori Norvell, DC Chapman (2021). Seeing the forest by looking at the trees: how to interpret a meta‐analysis forest plot. Global Spine J.

[BIBR-32] Thorlund K., Imberger G., Johnston B.C. (2012). Evolution of heterogeneity (I2) estimates and their 95% confidence intervals in large meta‐analyses. PLoS One.

[BIBR-33] Sterne J.A., Egger M. (2001). Funnel plots for detecting bias in meta‐analysis: guidelines on choice of axis. J Clin Epidemiol.

[BIBR-34] Begg C.B., Mazumdar M. (1994). Operating characteristics of a rank correlation test for publication bias. Biometrics.

[BIBR-35] Donohue J.F. (2005). Minimal clinically important differences in COPD lung function. COPD.

[BIBR-36] Crim C., Frith L.J., Midwinter D., Donohue J.F. (2021). FEV1 minimum important difference versus minimal detectable difference?. In search of the unicorn. Am J Respir Crit Care Med.

[BIBR-37] Kakavas S., Kotsiou O.S., Perlikos F. (2021). Pulmonary function testing in COPD: looking beyond the curtain of FEV1. NPJ Prim Care Respir Med.

[BIBR-38] Azhar N. (2015). Pre‐operative optimisation of lung function. Indian J Anaesth.

[BIBR-39] Belli S., Prince I., Savio G. (2021). Airway clearance techniques: the right choice for the right patient. Front Med (Lausanne.

[BIBR-40] Kim V., Criner G.J. (2013). Chronic bronchitis and chronic obstructive pulmonary disease. Am J Respir Crit Care Med.

[BIBR-41] Fuzhi Y., Dongfang T., Wentao F. (2022). Rapid recovery of postoperative pulmonary function in patients with lung cancer and influencing factors. Front Oncol.

[BIBR-42] Cukic V. (2014). Reduction of pulmonary function after surgical lung resections of different volume. Med Arch.

[BIBR-43] Alimy A.R., Polzer H., Ocokoljic A. (2023). Does minimally invasive surgery provide better clinical or radiographic outcomes than open surgery in the treatment of hallux valgus deformity? A systematic review and meta‐analysis. Clin Orthop Relat Res.

[BIBR-44] Hernández‐Vaquero D., Fernández‐Fairen M., Torres‐Perez A., Santamaría A. (2012). Minimally invasive surgery versus conventional surgery. A review of the scientific evidence [in Spanish. Rev Esp Cir Ortop Traumatol.

[BIBR-45] Campi R., Tellini R., Sessa F. (2019). European Society of Residents in Urology (ESRU). Techniques and outcomes of minimally‐invasive surgery for nonmetastatic renal cell carcinoma with inferior vena cava thrombosis: a systematic review of the literature. Minerva Urol Nefrol.

[BIBR-46] Yanik F. (2023). Current overview of awake, non‐intubated, video‐assisted thoracic surgery. Wideochir Inne Tech Maloinwazyjne.

[BIBR-47] Cukic V. (2012). Preoperative prediction of lung function in pneumonectomy by spirometry and lung perfusion scintigraphy. Acta Inform Med.

[BIBR-48] Chapkanov A., Todorova M., Chirlova A., Marinov B. (2024). Factors affecting prediction accuracy of postoperative FEV1 and DL,CO in patients undergoing lung resection. Folia Med (Plovdiv.

[BIBR-49] Galanis N., Farmakiotis D., Kouraki K., Fachadidou A. (2006). Forced expiratory volume in one second and peak expiratory flow rate values in non‐professional male tennis players. J Sports Med Phys Fitness.

[BIBR-50] Bujang M.A., Adnan T.H. (2016). Requirements for minimum sample size for sensitivity and specificity analysis. J Clin Diagn Res.

[BIBR-51] Papageorgiou C.V., Antoniou D., Kaltsakas G., Koulouris N.G. (2010). Role of quantitative CT in predicting postoperative FEV1 and chronic dyspnea in patients undergoing lung resection. Multidiscip Respir Med.

[BIBR-52] Kadkhodazadeh M., Shafizadeh M., Rahmatian M. (2022). Determination of the volume and density of mandibular ramus as a donor site using CBCT. J Maxillofac Oral Surg.

[BIBR-53] Kurzepa J., Bielewicz J., Czekajska‐Chehab E. (2011). CT Volume / density ratio as the marker of ischaemic brain injury. Acta Neurol Scand.

[BIBR-54] Underwood Anagnostopoulos, C Cerqueira (2004). British Cardiac Society; British Nuclear Cardiology Society; British Nuclear Medicine Society; Royal College of Physicians of London; Royal College of Radiologists. Myocardial perfusion scintigraphy: the evidence. Eur J Nucl Med Mol Imaging.

[BIBR-55] Iwano S., Okada T., Satake H., Naganawa S. (2009). 3D‐CT volumetry of the lung using multidetector row CT: comparison with pulmonary function tests. Acad Radiol.

[BIBR-56] Hong J.H., Goo J.M., Moon H.G. (2021). Usefulness of staging chest‐CT in patients with operable breast cancer. PLoS One.

[BIBR-57] Capitanio S., Nordin A.J., Noraini A.R., Rossetti C. (2016). PET/CT in nononcological lung diseases: current applications and future perspectives. Eur Respir Rev.

[BIBR-58] Moloney F., McWilliams S., Crush L. (2012). CT Densitometry as a predictor of pulmonary function in lung cancer patients. Open Respir Med J.

[BIBR-59] Stolk J., Versteegh M.I., Montenij L.J. (2007). Densitometry for assessment of effect of lung volume reduction surgery for emphysema. Eur Respir J.

[BIBR-60] Moloney F., McWilliams S., Crush L. (2012). CT densitometry as a predictor of pulmonary function in lung cancer patients. Open Respir Med J.

